# Molecular dynamics simulations in drug delivery research: Calcium chelation of G3.5 PAMAM dendrimers

**DOI:** 10.1080/23312009.2016.1229830

**Published:** 2016-09-22

**Authors:** David E. Jones, Albert M. Lund, Hamidreza Ghandehari, Julio C. Facelli

**Affiliations:** 1 Department of Biomedical Informatics, University of Utah, 421 Wakara, Salt Lake City, UT 84108, USA; 2 Department of Chemistry, University of Utah, Salt Lake City, UT 84112, USA; 3 Departments of Bioengineering and Pharmaceutics and Pharmaceutical Chemistry, University of Utah, Salt Lake City, UT 84112, USA; 4 Utah Center for Nanomedicine, Nano Institute of Utah, University of Utah, Salt Lake City, UT 84112, USA

**Keywords:** Computational and Theoretical Chemistry, Drug Design & Development, Drug Discovery, Nanobiotechnology, Oncology, Ca chelation, PAMAM dendrimers, MD simulations, nanomedicine

## Abstract

Poly(amido amine) (PAMAM) dendrimers have been considered as possible delivery systems for anticancer drugs. One potential advantage of these carriers would be their use in oral formulations, which will require absorption in the intestinal lumen. This may require the opening of tight junctions which may be enabled by reducing the Ca^2+^ concentration in the intestinal lumen, which has been shown as an absorption mechanism for EDTA (ethylenediaminetetraacetic acid). Using molecular dynamics simulations, we show that the G3.5 PAMAM dendrimers are able to chelate Ca^2+^ at similar proportions to EDTA, providing support to the hypothesis that oral formulations of PAMAM dendrimers could use this high chelating efficiency as a potential mechanism for permeating the tight junctions of the intestines if other formulation barriers could be overcome.

## 1. Introduction

Poly(amido amine) (PAMAM) dendrimers are complex molecules for which their biochemical activity *in vivo* is not fully understood. A particular mechanism of interest is the pathway by which orally taken PAMAM dendrimers could reach their target location when used as nanocarriers of anticancer drugs. Specifically, there is interest in understanding how these particles would be able to permeate the tight junctions of the intestinal lumen (Wang et al., 2010).

Literature consensus shows that tight junctions are dependent upon extracellular calcium (Ca^2+^) and magnesium for their integrity and function (Noach, Kurosaki, Blom-Roosemalen, & de Boer, 1993). Extracellular Ca^2+^ is responsible for keeping the tight junctions closed and it is known that lower concentrations of Ca^2+^ in the intestinal lumen lead to their opening. This has been clearly established using ethylenediaminetetraacetic acid (EDTA, Supplementary material Schema S1), a known Ca^2+^ chelator, that has been shown to open and transverse the tight junctions (Amsterdam & Jamieson, 1974). Several publications have suggested that PAMAM dendrimers also would be able to travel across the intestinal barrier using the same mechanism (Hubbard, Ghandehari, & Brayden, 2014; Hubbard et al., 2015). Due to their anionic charge the carboxylic acid (COOH) terminated Generation 3.5 (G3.5) PAMAM dendrimers may be capable of chelating Ca^2+^ in solution with a chelation efficiency similar to EDTA (Noach et al., 1993). Each COOH terminated G3.5 PAMAM dendrimer (Supplementary material Schema S2) can theoretically chelate 32 Ca^2+^ ions, thus significantly reducing the concentration of extracellular Ca^2+^ and therefore creating an extracellular environment that is prone to opening the tight junctions. This could allow for paracellular transport of G3.5 PAMAM dendrimers via the tight junctions. However, the ability of PAMAM dendrimers to chelate Ca^2+^ has yet to be confirmed by *in vitro* or *in vivo* studies. Simulation studies can be used to better understand this hypothesis and justify more experimental work.

Molecular dynamics (MD) simulations are routinely used to provide understanding and testing of novel hypotheses at the molecular scale for small molecule drug delivery research (Shinoda, DeVane, & Klein, 2012; Xiang & Anderson, 2006). With recent advances in computational power such simulations can now be used in nanomedicine, for instance, to better understand chemical and biological properties of PAMAM dendrimer nanoparticles. Many studies have focused on the use of MD simulations to gain insight on PAMAM dendrimer/ligand conformations and energies. A few molecular docking studies have been reported using MD simulations to analyze the interactions of siRNA and PAMAM dendrimers (Jensen et al., 2010; Karatasos, Posocco, Laurini, & Pricl, 2012; Vasumathi & Maiti, 2010). Other molecular docking studies have involved PAMAM dendrimers and ligands such as curcumin and porphyrin (Cao, Zhang, Wang, Yang, & Jiang, 2013; Castriciano et al., 2011). These include work by Avila-Salas et al. (2012) who used MD simulations and QSAR methods for *in silico* dendrimer–drug affinity studies. Ivanov and Jacobson (2008) used MD simulations to test the theoretical possibility of bivalent binding of a dendrimer, covalently appended with multiple copies of a ligand. Lee, Majoros, Williams, Athey, and Baker (2009) used MD simulations along with chemical analysis to guide the design of a multifunctional PAMAM dendrimer-based nanotherapeutic. Barata et al. (2011) used MD simulations to identify the key structural design principles for bioactive den-drimer molecules that could be synthesized and biologically evaluated. MD studies of PAMAM den-drimers chelation by various ions have been also recently reported (Astachov et al., 2016; Garzoni, Cheval, Fahmi, Danani, & Pavan, 2012), but to the authors’ knowledge no study has been performed on the chelating efficiency of Ca^2+^ by PAMAM dendrimers. In this article, we demonstrate how MD simulations can be used to test the chelating efficiency of G3.5 PAMAM dendrimers.

## 2. Methods

EDTA and G3.5 PAMAM dendrimer structures were manually constructed using MarvinSketch by ChemAxon (2013) and ChemAxon, Berry, and Ruyts (2012). The structures for each molecule were converted to their ionic forms by removing a hydrogen atom from the terminal COOH groups. This resulted in a minus four charge for the EDTA molecule, as there are four terminal COOH groups in it, and a minus 64 charge for the G3.5 PAMAM dendrimer molecule where there are 64 terminal COOH groups.

For all simulations, the ff12sb force fields were used along with the general AMBER force field (GAFF) to represent the EDTA and G3.5 PAMAM dendrimer (Case et al., 2014; Wang, Wang, Kollman, & Case, 2006). Four different solvent boxes were prepared for four different simulations: EDTA with Ca^2+^ in water, G3.5 PAMAM dendrimer with Ca^2+^ in water, EDTA with Ca^2+^ in a buffer solution, and G3.5 PAMAM with Ca^2+^ in the same buffer solution. The water model used in all the simulations was the TIP3P (Jorgensen, Chandrasekhar, Madura, Impey, & Klein, 1983). For both simulation studies in water, the concentration of Ca^2+^ was adjusted to approximately 0.115 M, while the simulation studies in the buffer solution were done using concentrations of Ca^2+^ ions of approximately 0.0575 M with the addition of sodium chloride (NaCl) at a concentration of approximately 0.115 M. The concentrations were calculated with respect to the volume of the simulation box and those used in the buffer simulations are representative of the experimental solutions used to examine the ability of EDTA to chelate Ca^2+^ and increase epithelial absorption by Vllasaliu et al. (2012) and Tomita, Hayashi, and Awazu (1996). The simulations in the buffer solution have been performed to further test the ability of Ca^2+^ chelation by EDTA and G3.5 PAMAM dendrimers, when a competing ion (Na^+^) is present in the solution. For the EDTA simulations the volume of the water box was approximately 75,000 Å^3^, while for the simulations with G3.5 PAMAM dendrimer the volume of the water box was approximately 460,000 Å^3^. All the simulations were performed using periodic boundary conditions and a non-bonded cutoff of 9.0 Å in the NPT ensemble. Specifically, the following number of molecules were used in the simulations: the EDTA and Ca^2+^ in water MD simulation has 1 EDTA molecule, 6 Cl^−^ ions, 5 Ca^2+^ ions, and 2,141 water molecules; the EDTA and Ca^2+^ in a buffer MD simulation has 1 EDTA molecule, 6 Na^+^ ions, 6 Cl^−^ ions, 2 Ca^2+^ ions, and 2,220 water molecules; the G3.5 PAMAM dendrimer and Ca^2+^ in water MD simulation has 1 G3.5 PAMAM dendrimer molecule, 32 Ca^2+^ ions, and 13,297 water molecules; the G3.5 PAMAM dendrimer and Ca^2+^ in a buffer MD simulation has 1 G3.5 PAMAM dendrimer molecule, 32 Na^+^ ions, 16 Ca^2+^ ions, and 13,035 water molecules.

Three independent MD simulations were performed for each system to obtain ensemble averages of multiple MD runs, which are those presented in the paper. The corresponding results for each of the simulations are provided in the supplementary material. Before each simulation all of the counter ions were randomly distributed in the water box at 8.00 Å from the molecule of interest, EDTA or G3.5 PAMAM dendrimer, using cpptraj (Roe & Cheatham, 2013).

The initial minimization step consisted of a total of 3,000 minimization iterations, of which 1,000 were done using the steepest descent method and 2,000 using conjugate gradients. Following the minimization, we performed a 40-ps equilibration phase where the temperature was increased from 0.0 to 300.0 K using 20,000 steps with a time step of 2 fs and using Langevin dynamics with a collision frequency of 1.0 ps^−1^. Following the equilibration, we performed a 30-ns MD simulation consisting of 1.5 × 10^7^ steps with a time step of 2 fs. During the MD simulation the temperature and pressure were held constant at 300.0 K and 1.0 bar, respectively, and Langevin dynamics was used with a collision frequency of 1.0 ps^−1^. The 30-ns MD simulation time was selected according to literature precedence (Vasumathi & Maiti, 2010) and the appropriateness of the selection was verified by the observation of the desired behavior (binding events strongly suggesting chelation) within the time frame, so no further simulation was necessary. Furthermore, comparison of the radial distributions at increasingly longer windows shows no significant differences (Supplementary material Figure S5).

Trajectory analysis of the 30-ns MD simulation was performed using cpptraj (Roe & Cheatham, 2013). The water molecules were removed from the trajectories and a custom Python script was used to plot the distance from each counter ion to the van der Waals surface of the molecule of interest (EDTA or G3.5 PAMAM dendrimer) for each time step of the 30-ns MD simulation. This script also calculated the average distance and minimum distance of each counter ion to the surface of the molecule of interest (EDTA or G3.5 PAMAM dendrimer) during the 30-ns MD simulation and the percentage of dwell time, that is the number of steps in which an individual counter ion is within 3.0 Å of the surface of the molecule divided by the total number of steps in the MD simulation.

The radial distribution functions were calculated within cpptraj for each set of counter ions (Ca^2+^, Na^+^, and Cl^−^) within each run of the MD simulations. The results reported here correspond to the average radial distributions over the three simulation runs performed for each system considered here (EDTA with Ca^2+^ in water, G3.5 PAMAM dendrimer with Ca^+2^ in water, EDTA with Ca^2+^ in a buffer solution, and G3.5 PAMAM with Ca^2+^ in the same buffer solution).

All calculations were performed on the Tangent cluster at the Center for High Performance Computing. Tangent is a part of the Adaptable Profile-Driven Testbed (Apt), an initiative by the Flux Research Group to provide a flexible, on-demand cloud computing environment targeted at researchers and scientists (https://www.flux.utah.edu/project/apt). We used our research project as a demonstration on how to use a computationally intensive application in a cloud computing environment. This study shows that it is possible to perform computationally significant drug delivery research in such an environment, which may provide researchers an appealing and more cost-effective alternative to traditional, dedicated high-performance computing (HPC) environments.

## 3. Results and discussion

As discussed above four different simulations were carried out: EDTA and Ca^2+^ in water, G3.5 PAMAM dendrimer and Ca^2+^ in water, EDTA and Ca^2+^ in a buffer, and G3.5 PAMAM dendrimer and Ca^2+^ in the same buffer. The results from each of these simulations as well as the performance of the Apt computational environment are described in the following subsections. In all cases our results are reported as an average of the values obtained in each of the three independent MD simulations performed for each system, to represent ensemble average values.

### 3.1. MD simulation study of EDTA and Ca^2+^ in water

**Figure 1:**
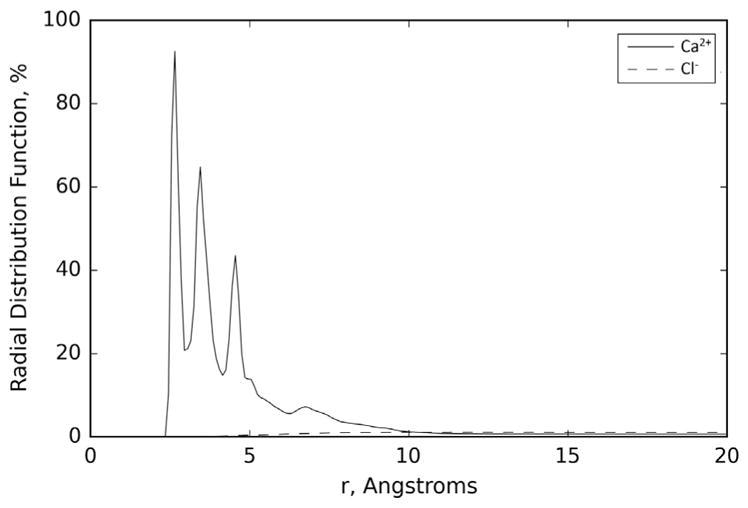
Average of the radial distributions of the Ca^2+^ and Cl^−^ ions over the three runs performed for the EDTA and Ca^2+^ system in water.

Results from the EDTA and Ca^2+^ in water MD simulations are presented in Figure 1 and Table 1. Figure 1 presents the radial distribution function of the Ca^2+^ and Cl^−^ ions in the EDTA and Ca^2+^ in water simulations, where it is apparent that the Ca^2+^ atoms are attached to the EDTA molecule. Table 1 shows the values of the averages of the distance, minimum distance, and percentage dwell time of the counter ions (Cl^−^ and Ca^2+^) included in the simulations. The average values and their standard deviations are the average over all the ions of the same type over all the trajectories from the three MD simulations performed here for this system.

Both the figure and table present results that are indicative of the ability of EDTA to chelate Ca^2+^ in water. As it can be seen in Table 1, the average minimum distance, 2.86 Å, from the surface of EDTA for the Ca^2+^ ions shows that in all the simulations the Ca^2+^ ions have migrated, at least temporarily, from a distance of 8.00 Å to a distance within 3.00 Å of the surface of the EDTA molecule. The average distance of the Ca^2+^ ions to the surface of the EDTA molecule is comparably large, but this is largely due to the fact that EDTA has only four terminal COOH groups. This allows for only two Ca^2+^ ions to bind to EDTA. To ensure that the same concentration of Ca^2+^ ions was used for all the MD simulation studies in water, five Ca^2+^ ions were used in the EDTA and Ca^2+^ in water MD simulations, therefore not all the Ca^2+^ ions can be in close proximity to the EDTA molecule, resulting in a larger average distance and standard deviation. This is also apparent from Figure 1, where the Ca^2+^ ions show secondary peaks corresponding to second and third absorption layers. The other two Ca^2+^ ions are at an average distance of ~2.5 Å, which corresponds to a situation in which the ions are bound to EDTA. This is confirmed by the average percentage dwell time for Ca^2+^ ions, which shows that for most of the simulation, 80% of the time, at least two of the Ca^2+^ ions are interacting with the surface of EDTA. These simulations’ results agree with the experimental results showing the large affinity of EDTA to chelate Ca^2+^ (Amsterdam & Jamieson, 1974).

The small standard deviations observed for the average minimum distance indicate that all ions at a given time in the simulation visit the proximity of the EDTA molecule, while the large standard deviations observed both for the average distance and average percentage dwell time of Ca^2+^ are an indication that there is dynamic equilibrium in which preferentially these ions are close to the EDTA surface. The small deviation on the Cl^−^ ion values indicates that these ions remain far from the EDTA surface; an expected result based on the EDTA and Cl^−^ polarity.

### 3.2. MD simulation study of EDTA and Ca^2+^ in a buffer solution

Results from the MD simulation study of EDTA and Ca^2+^ in a buffer are presented in Table 2, which shows the average distance, minimum distance, and average percentage dwell time of all the counter ions (Cl^−^, Na^+^, and Ca^2+^) in the buffer solution, as well as their standard deviations of these values when averaged over all the trajectories in the three simulations performed here for this system.

The average minimum distance of Ca^2+^ ions to the surface of the EDTA molecule is 2.54 Å, slightly smaller than the one observed in the water simulation and within 3.00 Å of the surface of the EDTA molecule. The average distance of the Ca^2+^ ions is 9.05 Å, also slightly smaller than in the water simulations. Its relatively large value can be explained by the same argument used for the simulations in water. The average dwell time for the Ca^2+^ ions also indicates that on average two Ca^2+^ ions are interacting with the surface of EDTA for a majority of the MD simulations. The results in the table show that the Na^+^ ions are much less likely to be close to the EDTA surface. It is not surprising that the minimum distance of Na^+^ (2.02 Å) is closer than that observed in the Ca^2+^, the Na^+^ ions are smaller and they only interact with one surface group on the EDTA molecule, whereas the Ca^2+^ ions commonly interact with two surface groups on the EDTA molecule. The average distance and the average percentage dwell time are considerably larger and smaller, respectively. These results show that while the Na^+^ is able to get in proximity of the EDTA, its interaction is less favorable than for Ca^2+^ as demonstrated by the larger average distance and smaller percentage dwell time. The corresponding radial distribution from this simulation (not shown) is similar to the one for the EDTA Ca^2+^ in water confirming the results reported in Table 2.

The results in the buffer solution are similar to those observed in the simulations of EDTA and Ca^2+^ in water. While it can be observed in the simulations that there is some competition for binding sites on EDTA between the six Na^+^ ions and two Ca^2+^ ions in the simulations, when comparing the results in Tables 1 and 2 it is apparent that this competition did not affect EDTA’s ability to chelate Ca^2+^. This indicates that the ability of EDTA to chelate Ca^2+^ is not affected by the presence of a competing ion (Na^+^) in the simulation environment. The results of the MD simulations are consistent with experimental results discussed above (Tomita et al., 1996; Vllasaliu et al., 2012).

The simulations results agree with the experimental results showing the ability of EDTA to chelate Ca^2+^ (Amsterdam & Jamieson, 1974) and validate our modeling approach to study chelation of G3.5 PAMAM dendrimers.

### 3.3. MD simulation study of G3.5 PAMAM dendrimer and Ca^2+^ in water

**Figure 2:**
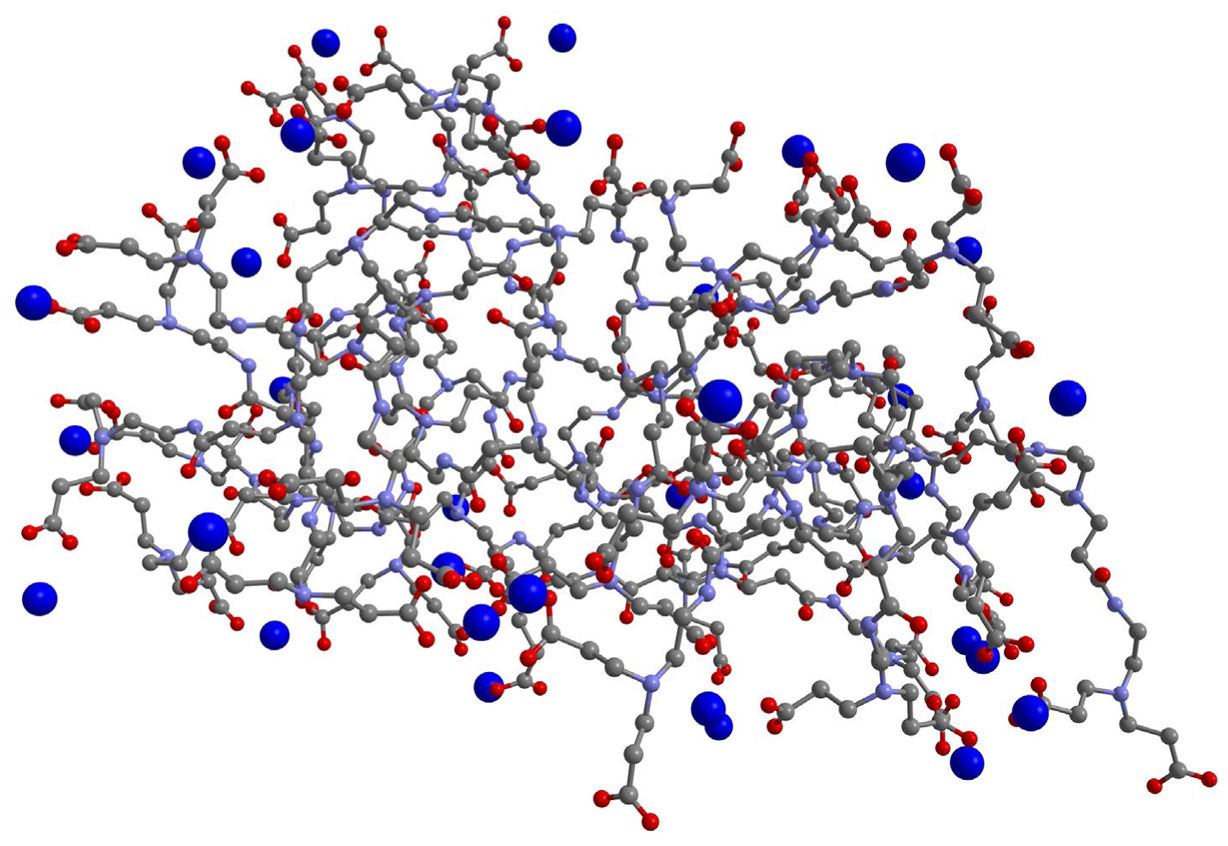
Three-dimensional representation of the final recorded step of one of the MD simulations of the G3.5 PAMAM dendrimer and Ca^2+^ in water. Note: The blue spheres represent Ca^2+^ ions present in close proximity to the G3.5 PAMAM dendrimer.

Results from the MD simulations of G3.5 PAMAM dendrimer and Ca^2+^ are presented in Figure 2 and Table 3. Figure 2 shows a three-dimensional depiction of the final recorded step of one of the simulations of G3.5 PAMAM dendrimer with Ca^2+^ in water, while Table 3 shows the values for the average distance and minimum distance from the surface of the G3.5 PAMAM dendrimer molecule and average percentage dwell time of the Ca^2+^ ions, as well as their standard deviations calculated over all the trajectories in the three independent simulations performed for this system.

Both the figure and table present results that clearly show the ability of G3.5 PAMAM dendrimer to chelate Ca^2+^. Figure 2 shows that multiple Ca^2+^ ions are interacting with the surface of the G3.5 PAMAM dendrimer molecule in the final recorded step of one of the MD simulation runs. As can be seen in Table 3, the average minimum distance of Ca^2+^ from the surface of G3.5 PAMAM dendrimer is 2.40 Å, which is closer than the average minimum distance of Ca^2+^ observed in the EDTA simulations in water (2.86 Å). Also, the average distance of the Ca^2+^ ions to the surface of the G3.5 PAMAM dendrimer molecule, 4.13 Å, is much smaller than that observed with EDTA (10.34 Å) in water consistent with the fact that in this system all Ca^2+^ ions can be bound to the G3.5 PAMAM at the same time. The average percentage dwell time of 0.86 shows that the G3.5 PAMAM dendrimer is nearly always, almost 90% of the time, binding Ca^2+^ ions. Note that the decrease in the average distance and dwell time is also associated with the fact that the calcium ions in this simulation are in the correct stoichiometric ratio, such that all the ions can be binding to the G3.5 PAMAM dendrimer at the same time.

### 3.4. MD simulation study of G3.5 PAMAM dendrimer and Ca^2+^ in a buffer solution

Results from the MD simulation study of the G3.5 PAMAM dendrimer and Ca^2+^ in a buffer are presented in Table 4 that shows the values for the average distance and minimum distance from the surface of the G3.5 PAMAM dendrimer molecule and the percentage dwell time of all the counter ions (Na^+^ and Ca^2+^) present in the buffer solution, as well as their standard deviations calculated over all trajectories from the three independent MD simulations performed in this system.

**Figure 3:**
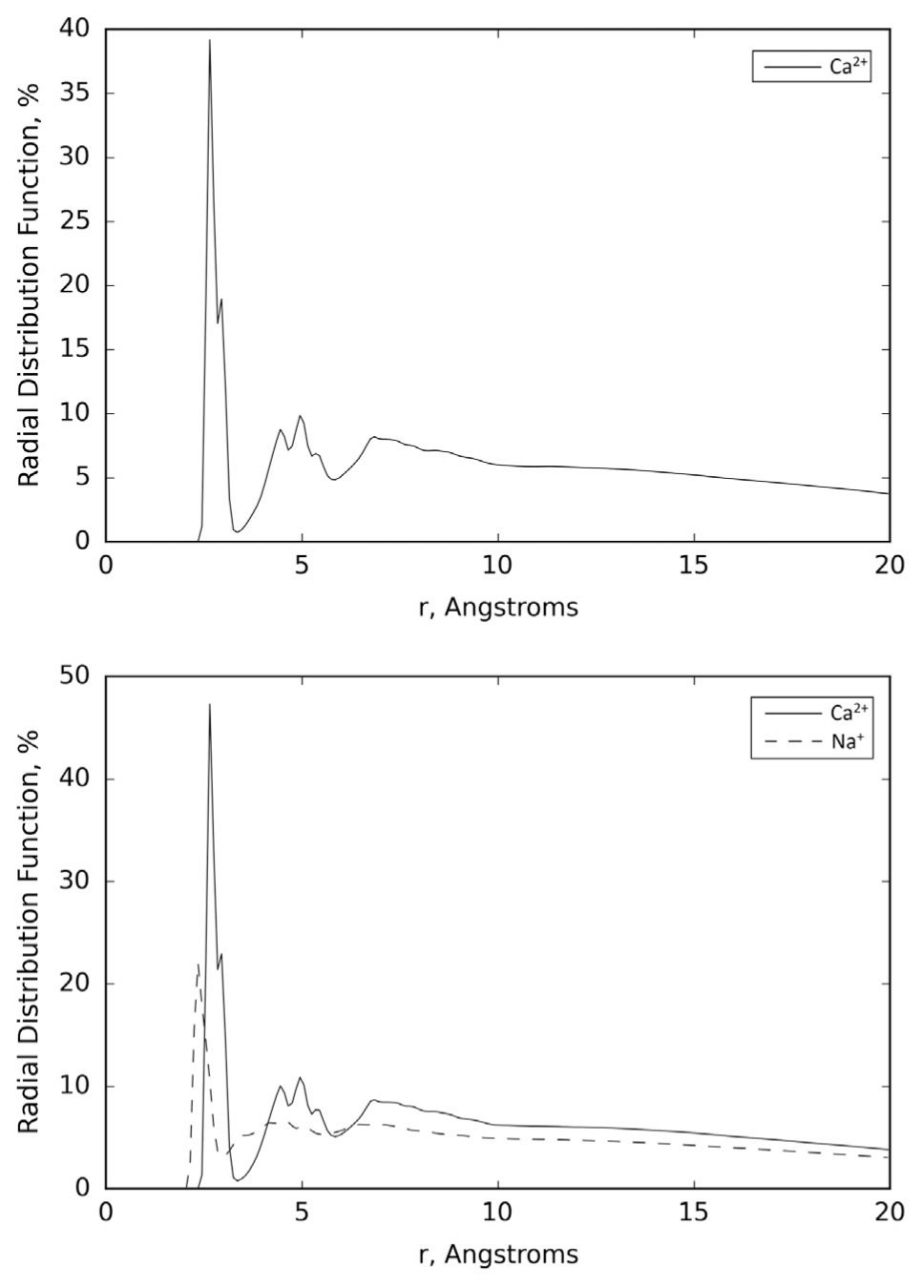
Average of the radial distribution functions of the Ca^2+^ ions over the three runs performed for the G3.5 PAMAM dendrimer and Ca^2+^ system in water (top) and the Ca^2+^ and Na^+^ ions over the three runs performed for the G3.5 PAMAM dendrimer in a buffer solution (bottom).

Figure 3 shows the average of the radial distribution functions of all the ions considered here over the three runs performed for the G3.5 PAMAM dendrimer and Ca^2+^ system in water and the G3.5 PAMAM dendrimer in a buffer solution. It is apparent from the figure that (i) all the Ca^2+^ ions are clearly attached to the G3.5 PAMAM dendrimer and (ii) that the introduction of potentially competitive Na^+^ ions does not change the chelation ability of Ca^2+^ ions by the G3.5 PAMAM dendrimers. The ability of Na^+^ to bind to the G3.5 PAMAM was independently verified by simulations without Ca^2+^ ions (supplementary material Table S5 and Figure S6).

These results show that the results obtained in water can also be expected in a buffer solution that mimics a situation closer to the intestinal lumen milieu and that the preferential binding is not sensitive to the details of the solution used in the simulations. Moreover, the preferential binding of Ca^2+^ is highlighted by the difference between the average distance of each ion to the surface of the G3.5 PAMAM. The average distance of the Ca^2+^ ions to the surface of the G3.5 PAMAM dendrimer molecule, 3.55 Å, is much smaller than that observed with the Na^+^ ions, 7.50 Å. This indicates that the Ca^2+^ ions appear to be more frequently bound to the G3.5 PAMAM dendrimer than the Na^+^ ions. The average minimum distance of Ca^2+^ from the surface of G3.5 PAMAM dendrimer of 2.40 Å is consistent with what was observed in the G3.5 PAMAM dendrimer and Ca^2+^ simulation in water.

The average minimum distance of Na^+^ (2.02 Å) is shorter than that observed in the Ca^2+^, in agreement with the results discussed above for the simulation of EDTA in buffer. Results of the average percentage dwell time of the Na^+^ and Ca^2+^ ions show a relatively large difference, 0.60 for Na^+^ and 0.91 for Ca^2+^ ions, which is similar to that observed in the simulation in water. This indicates that the Ca^2+^ ions appear to be more frequently bound to the G3.5 PAMAM dendrimer than the Na^+^ ions. Results show that Ca^2+^ is an effective chelation agent for G3.5 PAMAM dendrimer, indicating that under the simulation conditions the G3.5 dendrimer exhibits high chelating efficiency of Ca^2+^ in both water and in a buffer solution.

### 3.5. Apt computational environment

The use of AMBER in the Apt HPC environment has been extremely successful. During the course of these studies we were able to secure all the needed resources for the project, and because Apt is able to mirror a standard HPC environment, the migration to this cloud-based computing environment has been straightforward. Moreover, the overhead for using the Apt environment has been minimal. The only overhead incurred when using Apt is the instantiation and de-provisioning of the infrastructure, which is typically less than 15 min, a small fraction of a typical AMBER simulation.

## 4. Conclusions

Using MD simulations, validated by the agreement with existing experimental results in EDTA, we have shown that in MD simulations G3.5 PAMAM dendrimers exhibit a high chelating efficiency of Ca^2+^ comparable to the EDTA efficiency and therefore they may be capable of using this mechanism to open the tight junctions in the intestines. We have demonstrated that computationally intensive applications of interest in drug delivery research can effectively use cloud computing environments like Apt.

## Supplementary Material

Supplemental Materials

## Figures and Tables

**Table 1:** Average distance and average minimum distance from the van der Waals surface of the EDTA molecule and average percentage dwell time of the counter ions (Cl^−^ and Ca^2+^) included in this simulation

Counter ion	Average distance (Å)	Average minimum distance (Å)	Average (%) dwell time
Cl^−^	15.89 (0.47)	2.95 (0.21)	0.00 (0.0)
Ca^2+^	10.34 (5.70)	2.86 (0.58)	0.39 (0.42)

Note: Standard deviations, over the three independent runs of the EDTA and Ca^2+^ in water MD simulations, given between parentheses.

**Table 2:** Average distance and average minimum distance from the van der Waals surface of the EDTA molecule and average percentage dwell time of the counter ions (Cl^−^, Na^+^, and Ca^2+^) included in this simulation

Counter ion	Average distance (Å)	Average minimum distance (Å)	Average (%) dwell time
Cl^−^	15.84 (0.43)	3.05 (0.26)	0.00 (0.0)
Na^+^	12.26 (4.04)	2.02 (0.09)	0.24 (0.29)
Ca^2+^	9.05 (4.96)	2.54 (0.40)	0.47 (0.37)

Note: Standard deviations, over the three independent runs of the EDTA and Ca^2+^ in a buffer MD simulation, given between parentheses.

**Table 3:** Average distance and average minimum distance from the van der Waals surface of the G3.5 PAMAM molecule and average percentage dwell time of the counter ions (Ca^2+^) included in this simulation

Counter ion	Average distance (Å)	Average minimum distance (Å)	Average (%) dwell time
Ca^2+^	4.13 (2.83)	2.40 (0.02)	0.86 (0.22)

Note: Standard deviations, over the three independent runs of the G3.5 PAMAM dendrimer and Ca^2+^ in water MD simulation, given between parentheses.

**Table 4:** Average distance and average minimum distance from the van der Waals surface of the G3.5 PAMAM molecule and average percentage dwell time of the counter ions (Na^+^ and Ca^2+^) included in this simulation

Counter ion	Average distance (Å)	Average minimum distance (Å)	Average (%) dwell time
Na^+^	7.50 (3.13)	2.02 (0.05)	0.60 (0.20)
Ca^2+^	3.55 (2.20)	2.40 (0.01)	0.91 (0.13)

Note: Standard deviations, over the three independent runs of the G3.5 PAMAM dendrimer and Ca^2+^ in a buffer MD simulation, given between parentheses.
