# Supplementary material for: Molecular dynamics simulations in drug delivery research: Calcium chelation of G3.5 PAMAM dendrimers
Source: Cogent Chem. Author manuscript; Available in PMC 2017 Nov 22. (PMC5699217; doi:10.1080/23312009.2016.1229830)

Supplemental Information

Schema S1: Structure for EDTA.

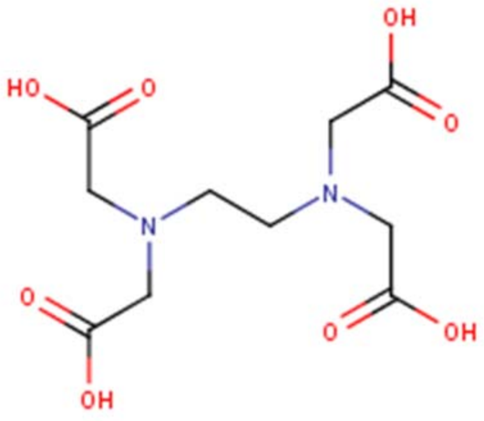

Schema S2: Structure for G3.5 PAMAM dendrimer
(R = $-NCH_2CH_2CONHCH_2CH_2NCH_2CH_2CONHCH_2CH_2NCH_2CH_2COOH$).

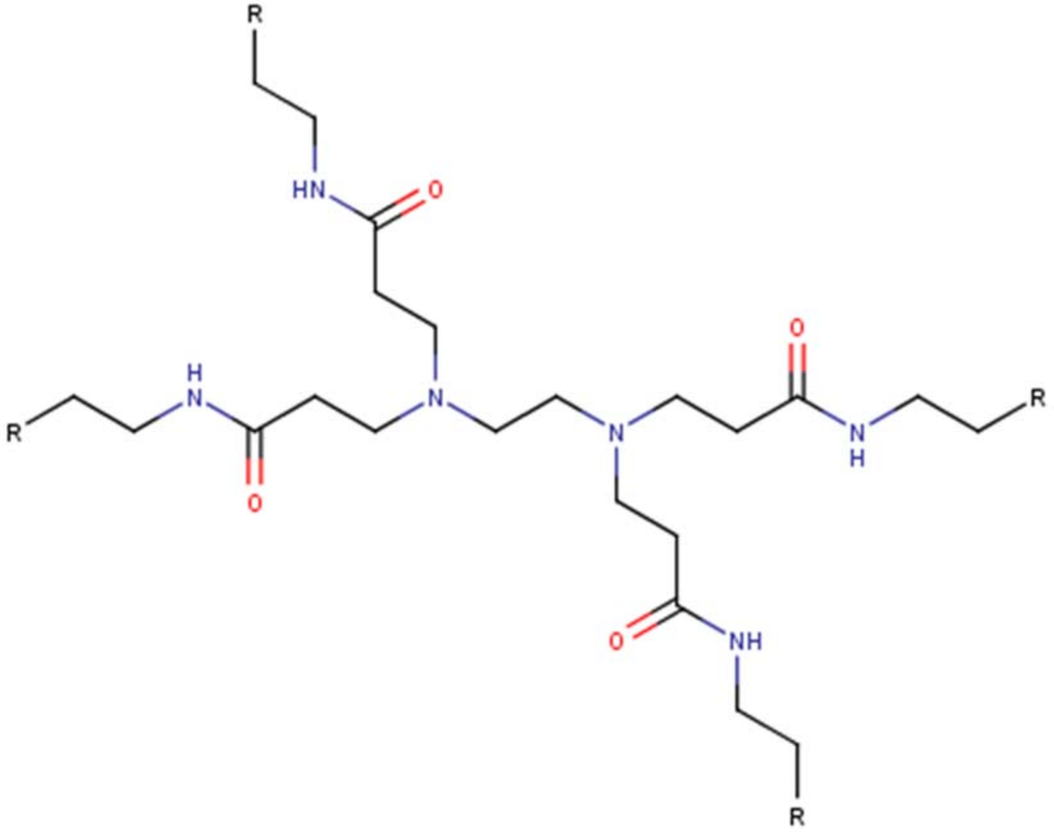

Table S1.1: Average distance and minimum distance from the van der Walls surface of the EDTA molecule and percentage dwell time of the counter ions (Cl^- and Ca^{2+}) included in the first EDTA and Ca^{2+} in water MD simulation.

Counter Ion	Average Distance (Å)	Minimum Distance (Å)	% Dwell Time
Cl^-	15.25	3.03	0
Cl^-	15.53	2.88	0
Cl^-	15.25	3.05	0
Cl^-	16.53	3.36	0
Cl^-	15.81	3.03	0
Cl^-	15.8	2.78	0
Ca^{2+}	4	2.35	0.81
Ca^{2+}	6.34	2.21	0.66
Ca^{2+}	16.51	3.79	0
Ca^{2+}	15.33	3.54	0
Ca^{2+}	7.58	2.34	0.65

Figure S1.1: The radial distributions of the counter ions (Cl^- and Ca^{2+}) included in the first EDTA and Ca^{2+} in water MD simulation.

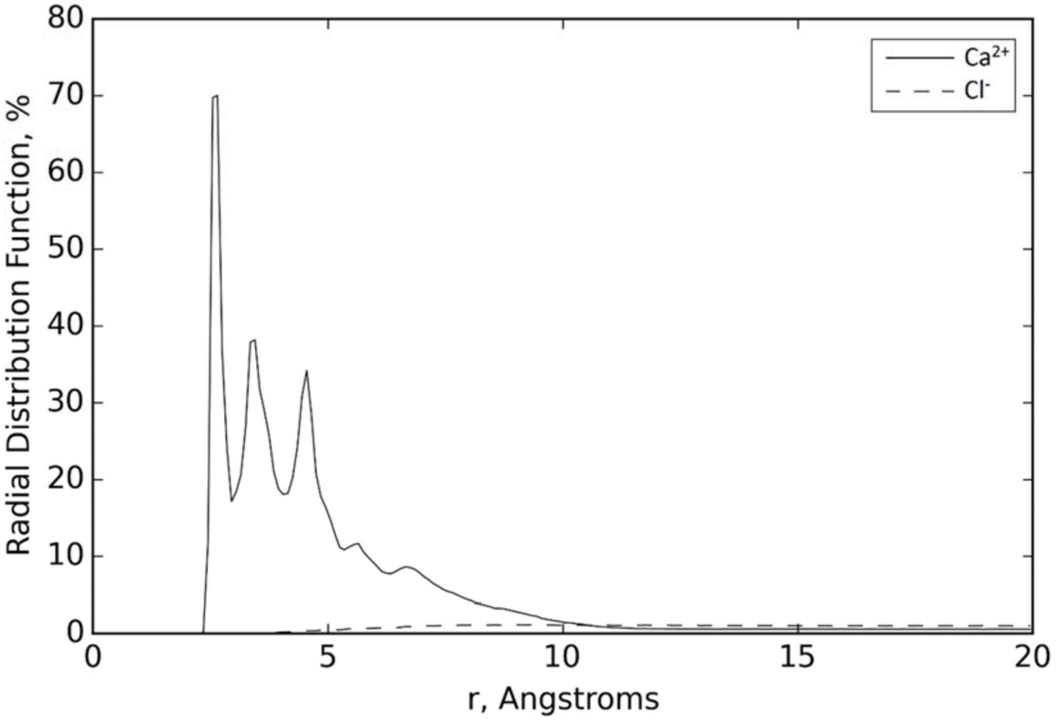

Table S1.2: Average distance and minimum distance from the van der Walls surface of the EDTA molecule and percentage dwell time of the counter ions (Cl^- and Ca^{2+}) included in the second EDTA and Ca^{2+} in water MD simulation.

Counter Ion	Average Distance (Å)	Minimum Distance (Å)	% Dwell Time
Cl^-	15.78	2.68	0
Cl^-	15.99	3.17	0
Cl^-	17.13	3.33	0
Cl^-	16.06	2.88	0
Cl^-	15.81	2.93	0
Cl^-	15.67	2.67	0
Ca^{2+}	2.8	2.38	0.95
Ca^{2+}	15.23	3.13	0
Ca^{2+}	15.43	3.42	0
Ca^{2+}	14.28	3.25	0
Ca^{2+}	5.17	2.35	0.81

Figure S1.2: The radial distributions of the counter ions (Cl^- and Ca^{2+}) included in the second EDTA and Ca^{2+} in water MD simulation.

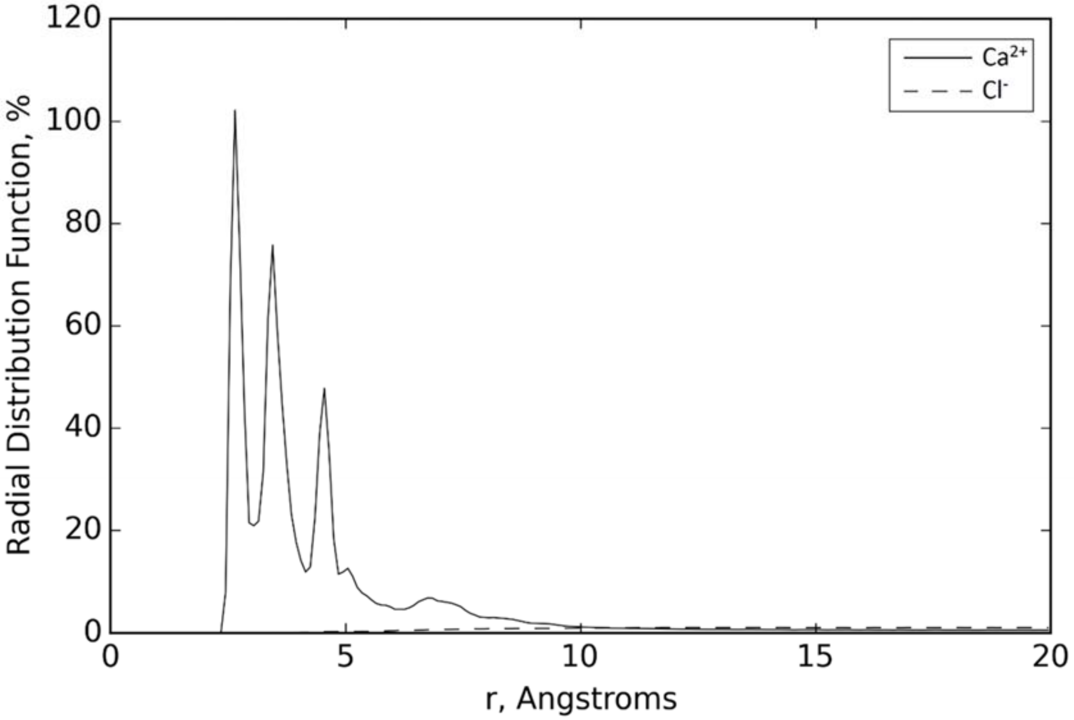

Table S1.3: Average distance and minimum distance from the van der Walls surface of the EDTA molecule and percentage dwell time of the counter ions (Cl^- and Ca^{2+}) included in the third EDTA and Ca^{2+} in water MD simulation.

Counter Ion	Average Distance (Å)	Minimum Distance (Å)	% Dwell Time
Cl^-	15.62	3.08	0
Cl^-	15.66	2.68	0
Cl^-	15.44	2.78	0
Cl^-	16.32	2.76	0
Cl^-	15.89	3.12	0
Cl^-	16.42	2.81	0
Ca^{2+}	16.52	3.35	0
Ca^{2+}	15.22	3.58	0
Ca^{2+}	14.25	2.43	0.17
Ca^{2+}	3.05	2.37	0.96
Ca^{2+}	3.32	2.35	0.88

Figure S1.3: The radial distributions of the counter ions (Cl^- and Ca^{2+}) included in the third EDTA and Ca^{2+} in water MD simulation.

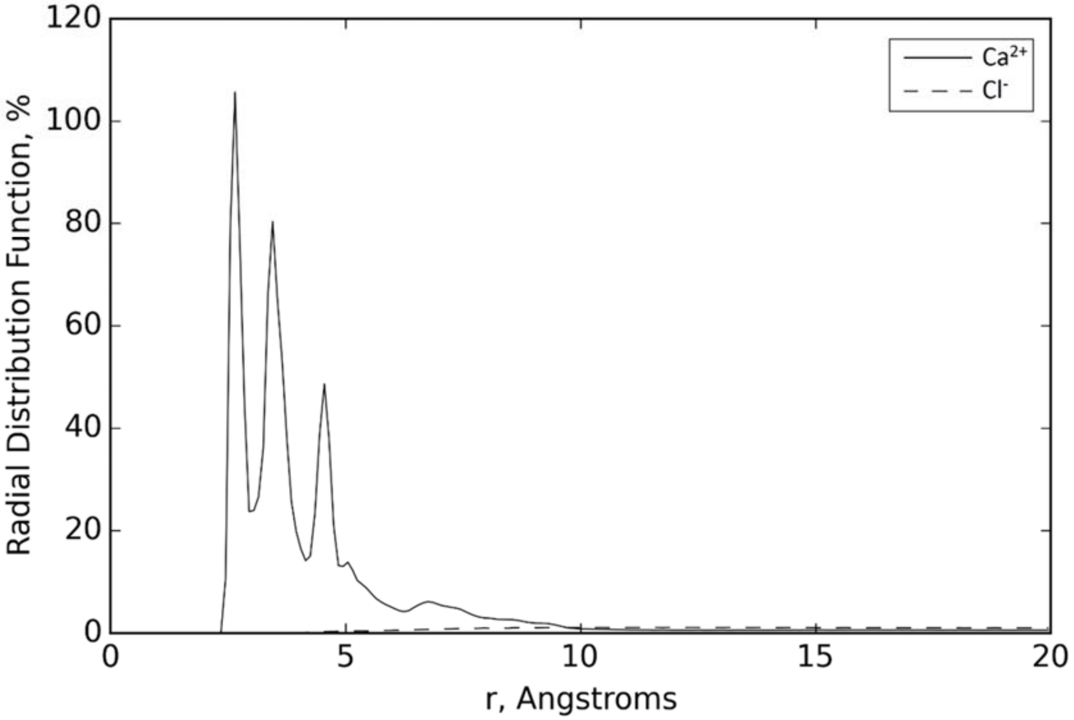

Table S2.1: Average distance and minimum distance from the van der Walls surface of the EDTA molecule and percentage dwell time of the counter ions (Cl^-, Na^+ and Ca^{2+}) included in the first EDTA and Ca^{2+} in a buffer MD simulation.

Counter Ion		Average Distance (Å)	Minimum Distance (Å)	% Dwell Time
Na^+		15.67	2.14	0.03
Na^+		16.87	2.22	0
Na^+		14.02	1.89	0.08
Na^+		8.8	1.9	0.5
Na^+		15.53	2.12	0.02
Na^+		14.23	1.95	0.09
Cl^-		15.89	3.83	0
Cl^-		16.32	2.77	0
Cl^-		16.3	3.24	0
Cl^-		15.95	3.14	0
Cl^-		16.02	3.14	0
Cl^-		15.95	2.81	0
Ca^{2+}		7.67	2.36	0.56
Ca^{2+}		5.11	2.32	0.79

Figure S2.1: The radial distributions of the counter ions (Cl^-, Na^+ and Ca^{2+}) included in the first EDTA and Ca^{2+} in a buffer MD simulation.

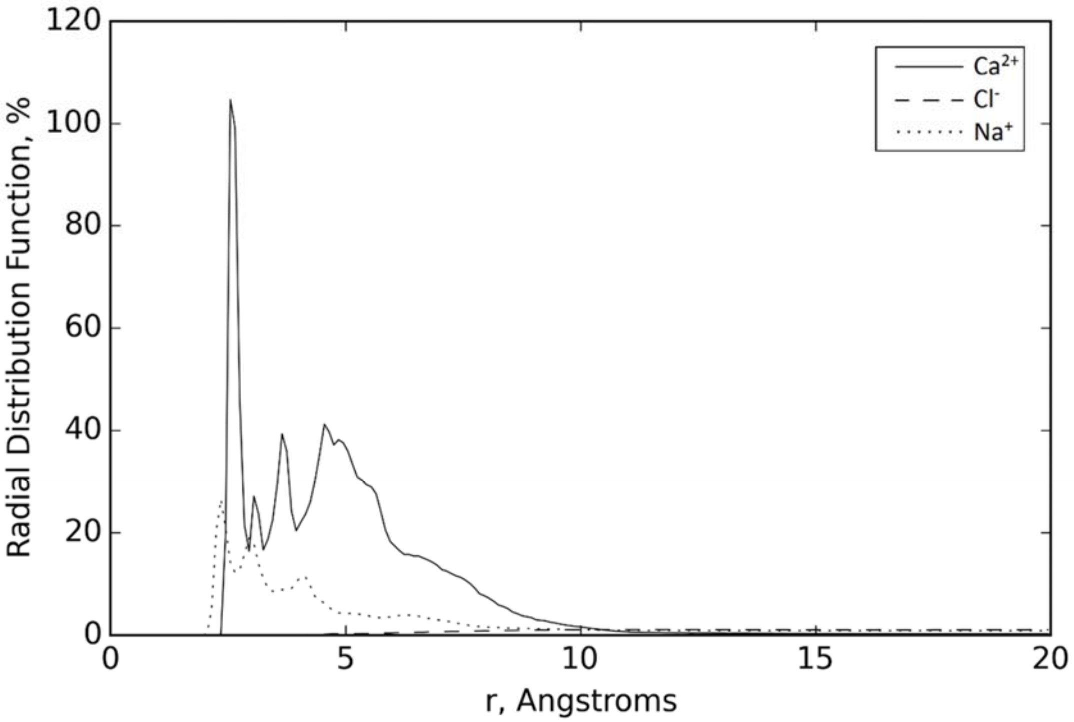

Table S2.2: Average distance and minimum distance from the van der Walls surface of the EDTA molecule and percentage dwell time of the counter ions (Cl^-, Na^+ and Ca^{2+}) included in the second EDTA and Ca^{2+} in a buffer MD simulation.

**Counter Ion**	**Average Distance (Å)**	**Minimum Distance (Å)**	**% Dwell Time**
Na^+	12.36	1.96	0.22
Na^+	13.57	2	0.2
Na^+	12.9	2	0.23
Na^+	11.79	1.99	0.23
Na^+	3.11	1.96	0.94
Na^+	16.75	2.03	0.01
Cl^-	15.73	2.85	0
Cl^-	16.21	2.79	0
Cl^-	16.14	2.76	0
Cl^-	16.06	3.06	0
Cl^-	15.82	3.14	0
Cl^-	16.32	3.29	0
Ca^{2+}	6.74	2.38	0.68
Ca^{2+}	15.78	2.44	0.01

Figure S2.2: The radial distributions of the counter ions (Cl^-, Na^+ and Ca^{2+}) included in the second EDTA and Ca^{2+} in a buffer MD simulation.

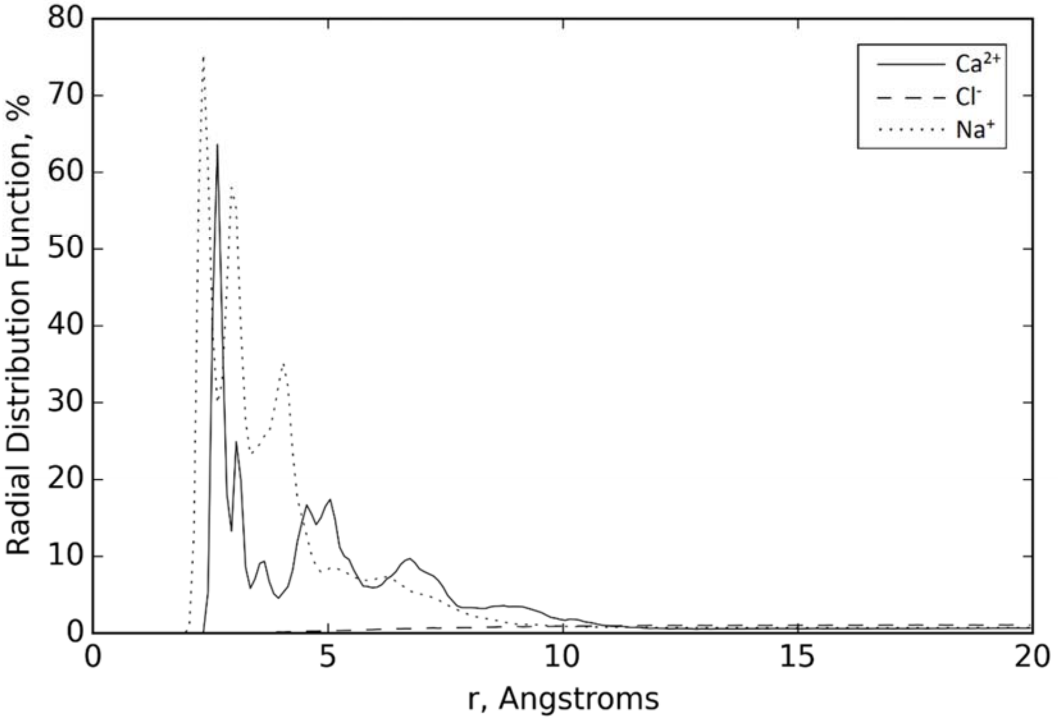

Table S2.3: Average distance and minimum distance from the van der Walls surface of the EDTA molecule and percentage dwell time of the counter ions (Cl^-, Na^+ and Ca^{2+}) included in the third EDTA and Ca^{2+} in a buffer MD simulation.

Counter Ion	Average Distance (Å)	Minimum Distance (Å)	% Dwell Time
Na^+	7.07	1.98	0.56
Na^+	14.82	2.13	0.01
Na^+	3.8	1.93	0.84
Na^+	12.02	1.99	0.23
Na^+	13.72	2.1	0.06
Na^+	13.67	2.06	0.05
Cl^-	15.44	3.01	0
Cl^-	15.38	3.03	0
Cl^-	15.54	3.15	0
Cl^-	14.57	2.74	0
Cl^-	15.93	3.13	0
Cl^-	15.55	3.06	0
Ca^{2+}	4.26	2.39	0.8
Ca^{2+}	14.71	3.36	0

Figure S2.3: The radial distributions of the counter ions (Cl^-**,** Na^+ and Ca^{2+}) included in the third EDTA and Ca^{2+} in a buffer MD simulation.

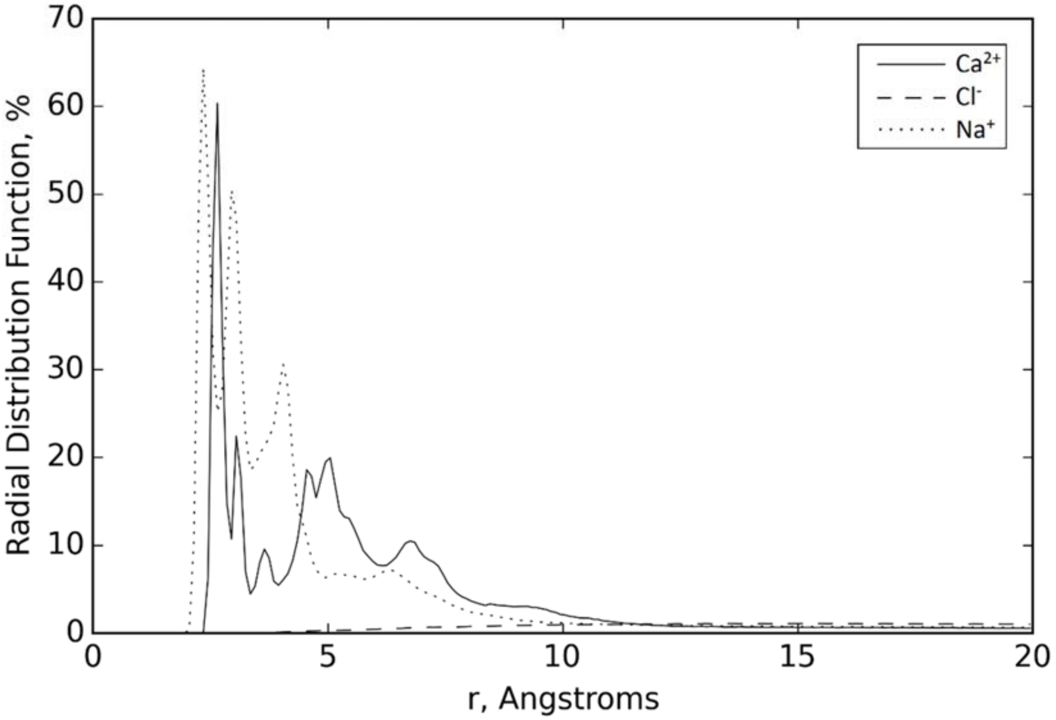

Table S3.1: Average distance and minimum distance from the van der Walls surface of the G3.5 PAMAM molecule and percentage dwell time of the counter ions (Ca^{2+}) included in the first G3.5 PAMAM dendrimer and Ca^{2+} in water MD simulation.

Counter Ion	Average Distance (Å)	Minimum Distance (Å)	% Dwell Time
Ca^{2+}	2.73	2.42	0.96
Ca^{2+}	3.88	2.41	0.88
Ca^{2+}	2.82	2.41	0.93
Ca^{2+}	9.25	2.41	0.61
Ca^{2+}	4.48	2.41	0.88
Ca^{2+}	3.04	2.41	0.95
Ca^{2+}	2.72	2.38	0.95
Ca^{2+}	3.31	2.38	0.95
Ca^{2+}	2.64	2.39	0.99
Ca^{2+}	7.9	2.41	0.52
Ca^{2+}	2.62	2.4	0.99
Ca^{2+}	2.85	2.4	0.93
Ca^{2+}	12.87	2.44	0.07
Ca^{2+}	2.7	2.39	0.99
Ca^{2+}	2.74	2.4	0.96
Ca^{2+}	10.66	2.43	0.52
Ca^{2+}	8.17	2.42	0.48
Ca^{2+}	3.29	2.41	0.93
Ca^{2+}	2.74	2.4	0.96
Ca^{2+}	2.82	2.4	0.97
Ca^{2+}	3.35	2.41	0.75
Ca^{2+}	2.79	2.4	0.98
Ca^{2+}	3.53	2.41	0.95
Ca^{2+}	3.79	2.39	0.9
Ca^{2+}	2.61	2.39	1
Ca^{2+}	2.99	2.38	0.96
Ca^{2+}	11.68	2.41	0.3
Ca^{2+}	2.88	2.4	0.96
Ca^{2+}	2.75	2.41	0.93

Ca^{2+}	2.64	2.38	0.99
Ca^{2+}	3.14	2.38	0.86
Ca^{2+}	3.73	2.41	0.92

Figure S3.1: The radial distributions of the counter ions (Ca^{2+}) included in the first G3.5 PAMAM dendrimer and Ca^{2+} in water MD simulation.

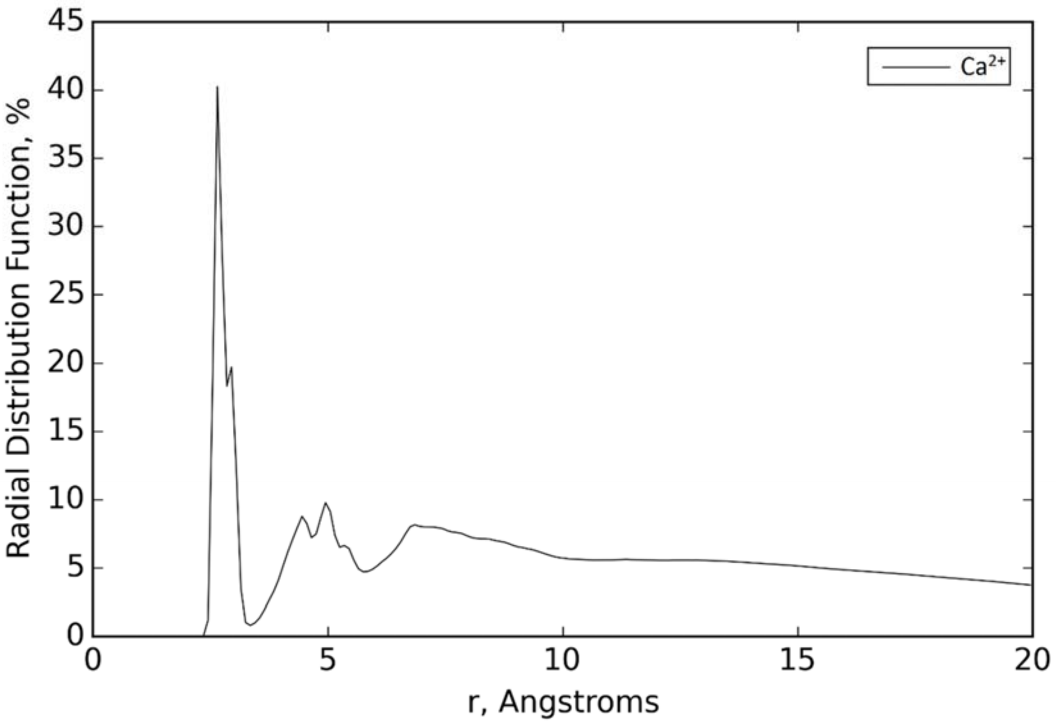

Table S3.2: Average distance and minimum distance from the van der Walls surface of the G3.5 PAMAM molecule and percentage dwell time of the counter ions (Ca^{2+}) included in the second G3.5 PAMAM dendrimer and Ca^{2+} in water MD simulation.

Counter Ion	Average Distance (Å)	Minimum Distance (Å)	% Dwell Time
Ca^{2+}	2.98	2.4	0.93
Ca^{2+}	11.97	2.42	0.38
Ca^{2+}	3.04	2.4	0.92
Ca^{2+}	3.36	2.4	0.94
Ca^{2+}	2.73	2.4	0.96
Ca^{2+}	14.66	2.49	0.02
Ca^{2+}	3.22	2.4	0.94
Ca^{2+}	2.79	2.39	0.92
Ca^{2+}	2.74	2.4	0.94
Ca^{2+}	4.18	2.41	0.89
Ca^{2+}	3.45	2.42	0.93
Ca^{2+}	2.64	2.39	0.98
Ca^{2+}	2.61	2.39	0.98
Ca^{2+}	2.63	2.41	0.99
Ca^{2+}	2.6	2.38	1
Ca^{2+}	2.65	2.39	0.98
Ca^{2+}	9.9	2.41	0.17
Ca^{2+}	2.75	2.4	0.98
Ca^{2+}	3.13	2.38	0.95
Ca^{2+}	2.62	2.41	0.99
Ca^{2+}	4.03	2.41	0.92
Ca^{2+}	2.87	2.4	0.94
Ca^{2+}	2.97	2.4	0.93
Ca^{2+}	7.22	2.41	0.65
Ca^{2+}	5.29	2.39	0.82
Ca^{2+}	13.34	2.44	0.32
Ca^{2+}	2.65	2.4	0.99
Ca^{2+}	2.9	2.39	0.98
Ca^{2+}	2.67	2.37	0.97

Ca^{2+}	2.6	2.36	1
Ca^{2+}	3.05	2.4	0.92
Ca^{2+}	2.83	2.4	0.97

Figure S3.2: The radial distributions of the counter ions (Ca^{2+}) included in the second G3.5 PAMAM dendrimer and Ca^{2+} in water MD simulation.

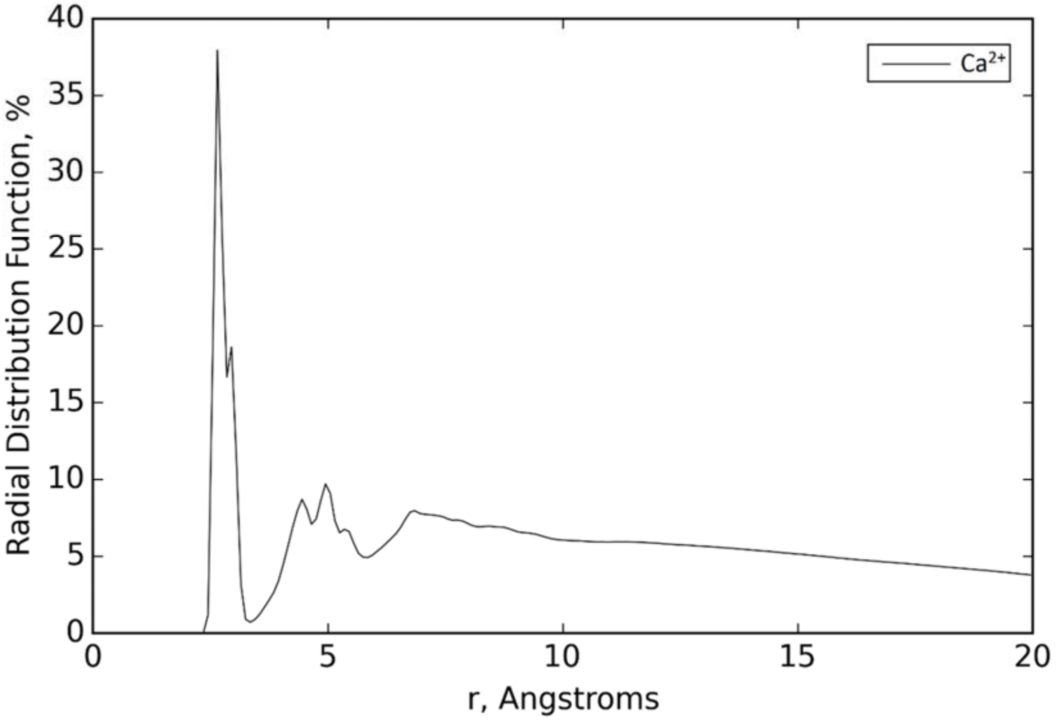

Table S3.3: Average distance and minimum distance from the van der Walls surface of the G3.5 PAMAM molecule and percentage dwell time of the counter ions (Ca^{2+}) included in the third G3.5 PAMAM dendrimer and Ca^{2+} in water MD simulation.

Counter Ion	Average Distance (Å)	Minimum Distance (Å)	% Dwell Time
Ca^{2+}	3.48	2.4	0.89
Ca^{2+}	3.97	2.38	0.77
Ca^{2+}	2.6	2.41	1
Ca^{2+}	3.18	2.41	0.88
Ca^{2+}	2.65	2.4	0.98
Ca^{2+}	2.61	2.4	0.99
Ca^{2+}	2.88	2.4	0.91
Ca^{2+}	2.64	2.4	0.99
Ca^{2+}	10.97	2.42	0.4
Ca^{2+}	4.33	2.4	0.77
Ca^{2+}	2.95	2.38	0.97
Ca^{2+}	2.71	2.42	0.95
Ca^{2+}	2.64	2.38	0.98
Ca^{2+}	3.64	2.43	0.68
Ca^{2+}	2.59	2.39	0.99
Ca^{2+}	2.67	2.38	0.97
Ca^{2+}	5.94	2.39	0.77
Ca^{2+}	3.66	2.39	0.79
Ca^{2+}	3.01	2.39	0.96
Ca^{2+}	2.6	2.41	0.99
Ca^{2+}	2.81	2.39	0.98
Ca^{2+}	3.26	2.4	0.95
Ca^{2+}	3.13	2.39	0.96
Ca^{2+}	3.26	2.4	0.85
Ca^{2+}	2.76	2.41	0.98
Ca^{2+}	11.82	2.4	0.26
Ca^{2+}	4	2.42	0.84
Ca^{2+}	2.74	2.38	0.99
Ca^{2+}	2.73	2.39	0.96

Ca^{2+}	2.76	2.38	0.91
Ca^{2+}	3.99	2.39	0.81
Ca^{2+}	2.94	2.4	0.92

Figure S3.3: The radial distributions of the counter ions (Ca^{2+}) included in the third G3.5 PAMAM dendrimer and Ca^{2+} in water MD simulation.

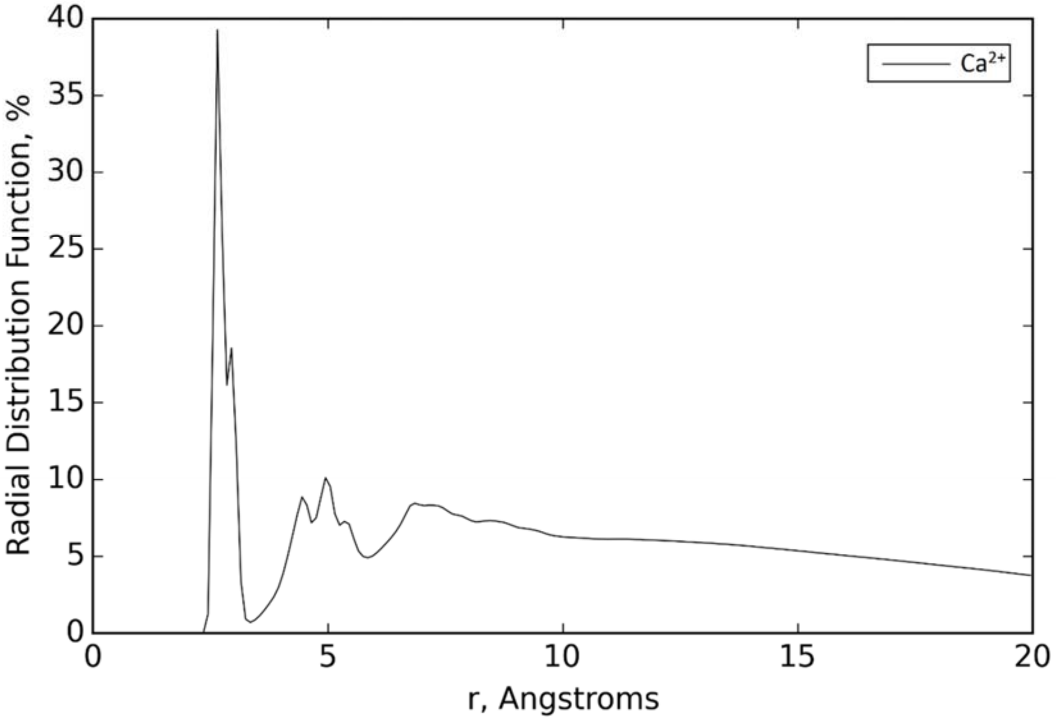

Table S4.1: Average distance and minimum distance from the van der Walls surface of the G3.5 PAMAM molecule and percentage dwell time of the counter ions (Na^+ and Ca^{2+}) included in the first G3.5 PAMAM dendrimer and Ca^{2+} in a buffer MD simulation.

Counter Ion	Average Distance (Å)	Minimum Distance (Å)	% Dwell Time
Na^+	9.89	2.04	0.56
Na^+	7.09	2.02	0.62
Na^+	10.3	1.99	0.53
Na^+	7.32	2.06	0.56
Na^+	3.61	2	0.84
Na^+	7.52	1.88	0.61
Na^+	10.93	2.02	0.42
Na^+	9.97	2.03	0.46
Na^+	9.77	2.09	0.4
Na^+	10.88	2.05	0.39
Na^+	7.89	1.93	0.49
Na^+	4.36	2.03	0.8
Na^+	7.55	2.04	0.46
Na^+	8.14	2.08	0.51
Na^+	4.83	2.04	0.8
Na^+	8.18	2.03	0.58
Na^+	2.48	2.05	0.95
Na^+	3.78	1.92	0.85
Na^+	5.68	1.97	0.7
Na^+	9.3	2.04	0.47
Na^+	4.8	2.01	0.74
Na^+	2.44	2.01	0.95
Na^+	3.49	2.06	0.82
Na^+	16.16	2.1	0.12
Na^+	7.02	2.05	0.52
Na^+	2.76	1.98	0.94
Na^+	14.94	2.05	0.11
Na^+	10.86	2.02	0.48
Na^+	6.47	2.03	0.58

Na^{+}	5.87	1.95	0.75
Na^{+}	10.96	2.01	0.35
Na^{+}	9.73	2.09	0.52
Ca^{2+}	2.68	2.4	0.96
Ca^{2+}	13.04	2.4	0.36
Ca^{2+}	7.13	2.4	0.75
Ca^{2+}	3.17	2.41	0.83
Ca^{2+}	2.64	2.38	0.97
Ca^{2+}	2.6	2.39	0.98
Ca^{2+}	2.61	2.39	0.99
Ca^{2+}	3.01	2.38	0.97
Ca^{2+}	2.62	2.4	0.99
Ca^{2+}	2.59	2.39	0.99
Ca^{2+}	2.84	2.4	0.98
Ca^{2+}	2.68	2.41	0.95
Ca^{2+}	2.83	2.39	0.94
Ca^{2+}	2.61	2.39	0.99
Ca^{2+}	2.75	2.39	0.97
Ca^{2+}	3.85	2.39	0.88

Figure S4.1: The radial distributions of the counter ions (Na^+ and Ca^{2+}) included in the first G3.5 PAMAM dendrimer and Ca^{2+} in a buffer MD simulation.

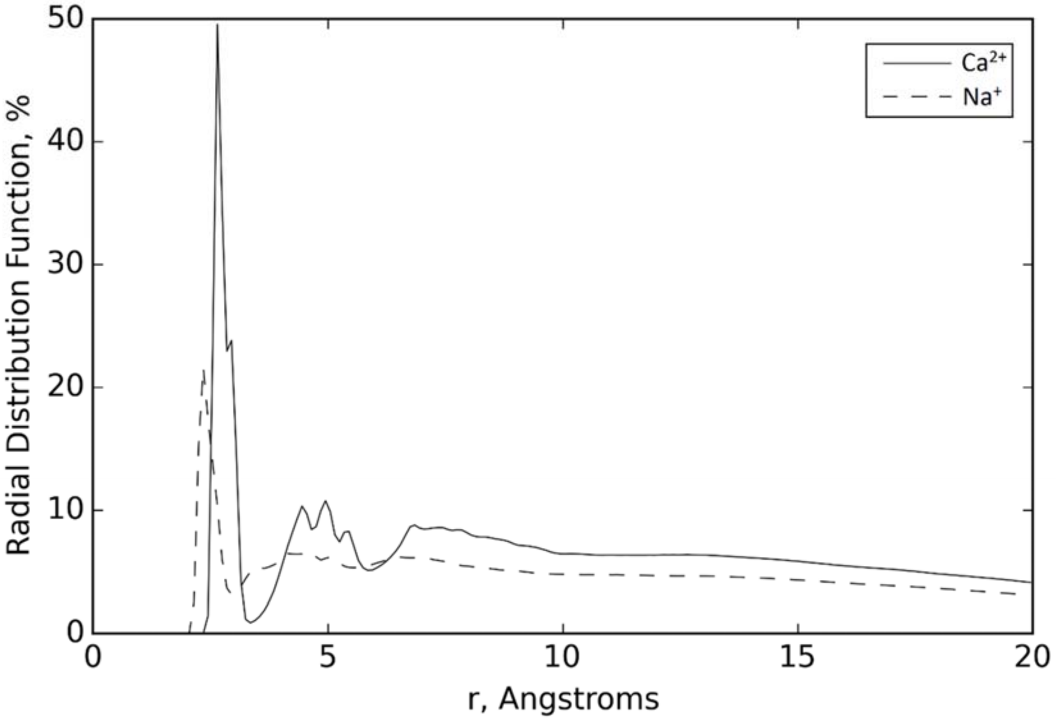

Table S4.2: Average distance and minimum distance from the van der Walls surface of the G3.5 PAMAM molecule and percentage dwell time of the counter ions (Na^+ and Ca^{2+}) included in the second G3.5 PAMAM dendrimer and Ca^{2+} in a buffer MD simulation.

Counter Ion	Average Distance (Å)	Minimum Distance (Å)	% Dwell Time
Na^+	7.01	2.04	0.62
Na^+	9.69	1.93	0.44
Na^+	5.74	1.98	0.68
Na^+	7.27	2.05	0.71
Na^+	10.76	2	0.35
Na^+	3.95	1.96	0.84
Na^+	9.78	1.99	0.49
Na^+	8.25	2.05	0.57
Na^+	7.95	2.07	0.49
Na^+	8.68	2.07	0.55
Na^+	6.75	2.05	0.76
Na^+	2.54	2.04	0.95
Na^+	7.08	2.02	0.6
Na^+	8.44	2.09	0.5
Na^+	6.13	2.06	0.72
Na^+	5.31	2.06	0.66
Na^+	10.8	2.04	0.33
Na^+	7.84	2.04	0.71
Na^+	2.5	2.02	0.98
Na^+	5.31	2.03	0.69
Na^+	5.39	2	0.72
Na^+	10.32	2.05	0.38
Na^+	5.07	2.03	0.77
Na^+	13.6	2.04	0.23
Na^+	8.93	2.01	0.53
Na^+	10.46	1.97	0.32
Na^+	3.62	1.95	0.83
Na^+	6.4	1.98	0.76
Na^+	7.33	2.06	0.51

Na^+	10.39	2.07	0.45
Na^+	8.65	2.07	0.52
Na^+	9.52	1.97	0.57
Ca^{2+}	2.77	2.4	0.98
Ca^{2+}	2.83	2.41	0.93
Ca^{2+}	8	2.39	0.64
Ca^{2+}	2.65	2.4	0.98
Ca^{2+}	2.88	2.4	0.95
Ca^{2+}	5.78	2.39	0.75
Ca^{2+}	4.39	2.4	0.66
Ca^{2+}	2.7	2.42	0.99
Ca^{2+}	2.64	2.39	0.99
Ca^{2+}	2.66	2.4	0.98
Ca^{2+}	2.88	2.4	0.98
Ca^{2+}	2.9	2.4	0.95
Ca^{2+}	3.11	2.41	0.95
Ca^{2+}	2.76	2.42	0.94
Ca^{2+}	3.03	2.4	0.96
Ca^{2+}	3.31	2.4	0.84

Figure S4.2: The radial distributions of the counter ions (Na^+ and Ca^{2+}) included in the second G3.5 PAMAM dendrimer and Ca^{2+} in a buffer MD simulation.

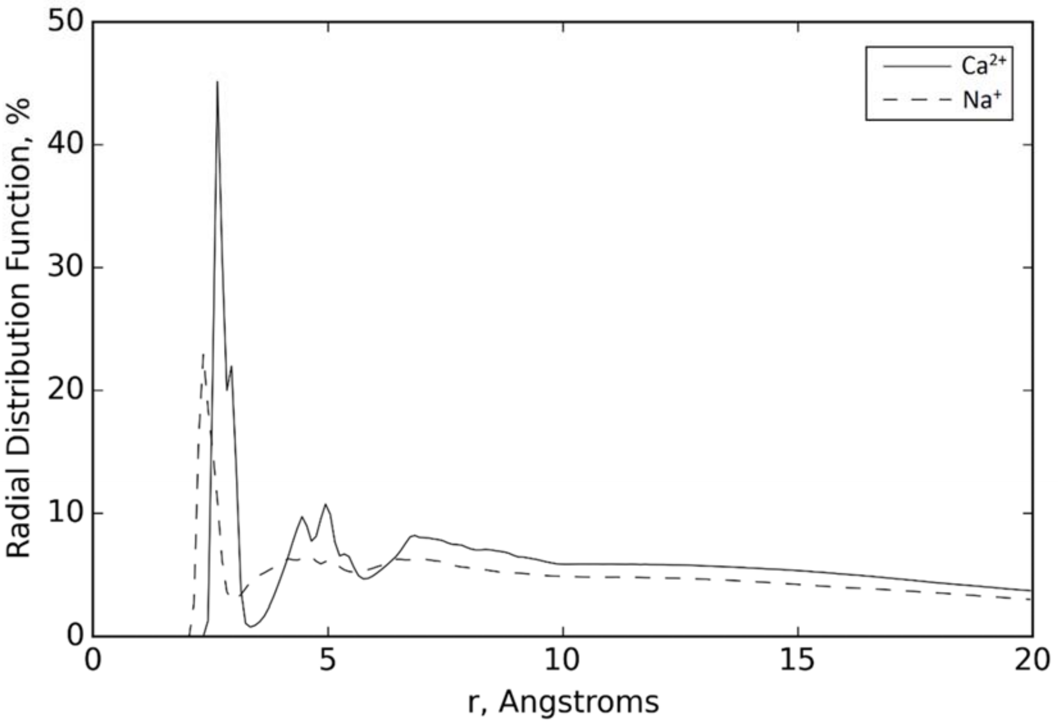

Table S4.3: Average distance and minimum distance from the van der Walls surface of the G3.5 PAMAM molecule and percentage dwell time of the counter ions (Na^+ and Ca^{2+}) included in the third G3.5 PAMAM dendrimer and Ca^{2+} in a buffer MD simulation.

Counter Ion	Average Distance (Å)	Minimum Distance (Å)	% Dwell Time
Na^+	7.22	1.98	0.62
Na^+	2.94	2.05	0.9
Na^+	8.54	1.97	0.54
Na^+	6.04	2.06	0.54
Na^+	9.22	1.97	0.59
Na^+	7.69	2.03	0.55
Na^+	3.55	2.07	0.77
Na^+	2.52	1.93	0.96
Na^+	8.09	2.06	0.45
Na^+	11.09	1.97	0.34
Na^+	6.15	2.02	0.75
Na^+	5.3	2.01	0.64
Na^+	6.7	2.01	0.6
Na^+	15.69	2.05	0.23
Na^+	14.49	2.09	0.11
Na^+	8.92	1.97	0.55
Na^+	2.28	2.03	1
Na^+	4.27	2.07	0.83
Na^+	2.69	1.96	0.88
Na^+	7.23	1.94	0.63
Na^+	9.22	2.06	0.48
Na^+	11.24	2.02	0.42
Na^+	5.78	2.08	0.67
Na^+	9.07	2.07	0.57
Na^+	9.26	2	0.46
Na^+	2.39	1.92	0.95
Na^+	10.08	1.92	0.38
Na^+	4.66	1.91	0.78
Na^+	6.65	2.02	0.57

Na^{+}	11.64	2	0.36
Na^{+}	8.41	1.99	0.43
Na^{+}	5.01	2.05	0.78
Ca^{2+}	2.79	2.4	0.94
Ca^{2+}	2.59	2.4	1
Ca^{2+}	12.24	2.41	0.46
Ca^{2+}	2.61	2.4	0.99
Ca^{2+}	2.82	2.4	0.98
Ca^{2+}	3.27	2.38	0.9
Ca^{2+}	2.76	2.4	0.98
Ca^{2+}	3.08	2.41	0.89
Ca^{2+}	2.69	2.4	0.95
Ca^{2+}	2.66	2.41	0.99
Ca^{2+}	2.86	2.4	0.95
Ca^{2+}	2.75	2.39	0.97
Ca^{2+}	3.02	2.41	0.97
Ca^{2+}	3.24	2.41	0.86
Ca^{2+}	3.03	2.41	0.94
Ca^{2+}	2.93	2.41	0.86

Figure S4.3: The radial distributions of the counter ions (Na^+ and Ca^{2+}) included in the third G3.5 PAMAM dendrimer and Ca^{2+} in a buffer MD simulation.

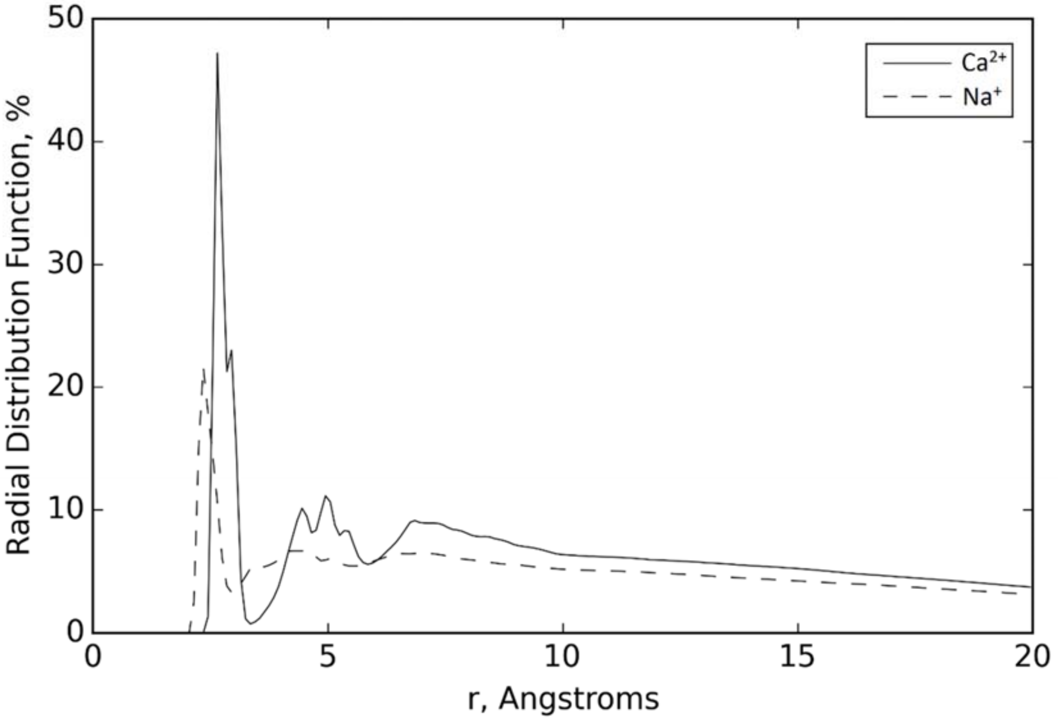

Figure S5: The radial distributions of the counter ions (Ca^{2+}) included in the G3.5 PAMAM dendrimer and Ca^{2+} in water MD simulation shown for three different time intervals (0-5 ns, 0-10 ns, and 0-15 ns) to show that equilibrium was established.

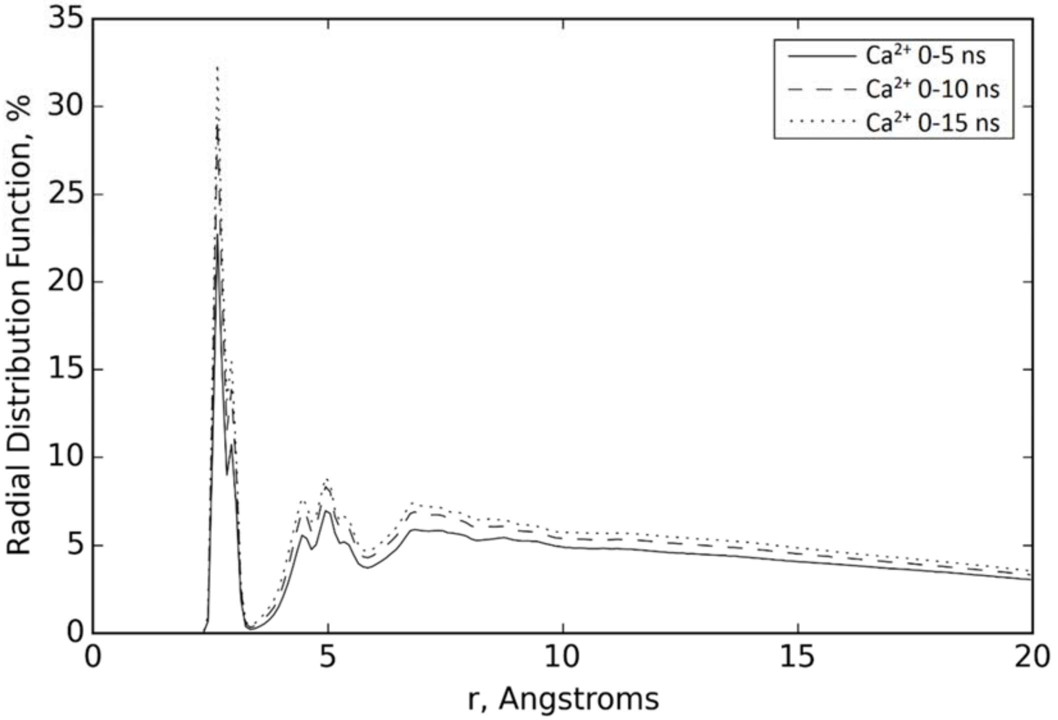

Table S5: Average distance and minimum distance from the van der Walls surface of the G3.5 PAMAM molecule and percentage dwell time of the counter ions (Na^+) included in the third G3.5 PAMAM dendrimer and Na^+ in water MD simulation.

Counter Ion	Average Distance (Å)	Minimum Distance (Å)	% Dwell Time
Na^+	4.39	2.06	0.7
Na^+	2.41	2	0.96
Na^+	4.65	1.98	0.74
Na^+	4.07	2.01	0.82
Na^+	3.66	2	0.83
Na^+	6.31	1.96	0.67
Na^+	7.02	2.01	0.51
Na^+	5.1	2.03	0.76
Na^+	5.23	2	0.65
Na^+	6.78	2.05	0.63
Na^+	2.31	2.06	0.99
Na^+	5.23	2.05	0.71
Na^+	5.3	2.07	0.71
Na^+	5.02	1.96	0.65
Na^+	3.11	2.03	0.85
Na^+	11	2.06	0.32
Na^+	8.55	2.05	0.52
Na^+	3.05	2	0.82
Na^+	3.12	1.97	0.86
Na^+	7.11	2.06	0.56
Na^+	7.11	2.06	0.56
Na^+	2.84	2.05	0.88
Na^+	4.83	2.07	0.72
Na^+	5.9	2.01	0.64
Na^+	4.14	1.99	0.7
Na^+	4.48	2	0.79

Na^+	9.19	2.02	0.41
Na^+	2.33	1.9	0.97
Na^+	10.64	2.08	0.32
Na^+	2.36	2.05	0.96
Na^+	12.61	2.09	0.27
Na^+	6.63	2.07	0.56
Na^+	9.73	2.01	0.32
Na^+	3.3	2	0.86
Na^+	7.44	1.99	0.56
Na^+	9	2.09	0.42
Na^+	4.09	2.06	0.73
Na^+	10.01	2.01	0.43
Na^+	4.31	2.07	0.79
Na^+	6.69	2.07	0.54
Na^+	10.09	2.07	0.26
Na^+	2.89	1.93	0.9
Na^+	3.5	1.93	0.83
Na^+	6.57	2.05	0.63
Na^+	5.75	2.05	0.67
Na^+	3.31	1.94	0.85
Na^+	14.37	2.09	0.2
Na^+	10.77	2.07	0.28
Na^+	2.88	2.05	0.92
Na^+	4.73	2.08	0.62
Na^+	5.74	2.01	0.7
Na^+	3.15	2.01	0.86
Na^+	2.6	1.93	0.93
Na^+	6.22	1.99	0.59
Na^+	4.2	2.03	0.82
Na^+	5.44	2	0.64
Na^+	3.28	1.95	0.89
Na^+	3.11	2.03	0.89
Na^+	10.28	2.1	0.33
Na^+	3.01	2.05	0.84
Na^+	8.45	2.09	0.41

Na^+	11.66	2.08	0.26
Na^+	7.1	2.02	0.59
Na^+	5.22	2.05	0.64

Figure S6: The radial distributions of the counter ions (Na^+) included in the third G3.5 PAMAM dendrimer and Na^+ in water MD simulation.

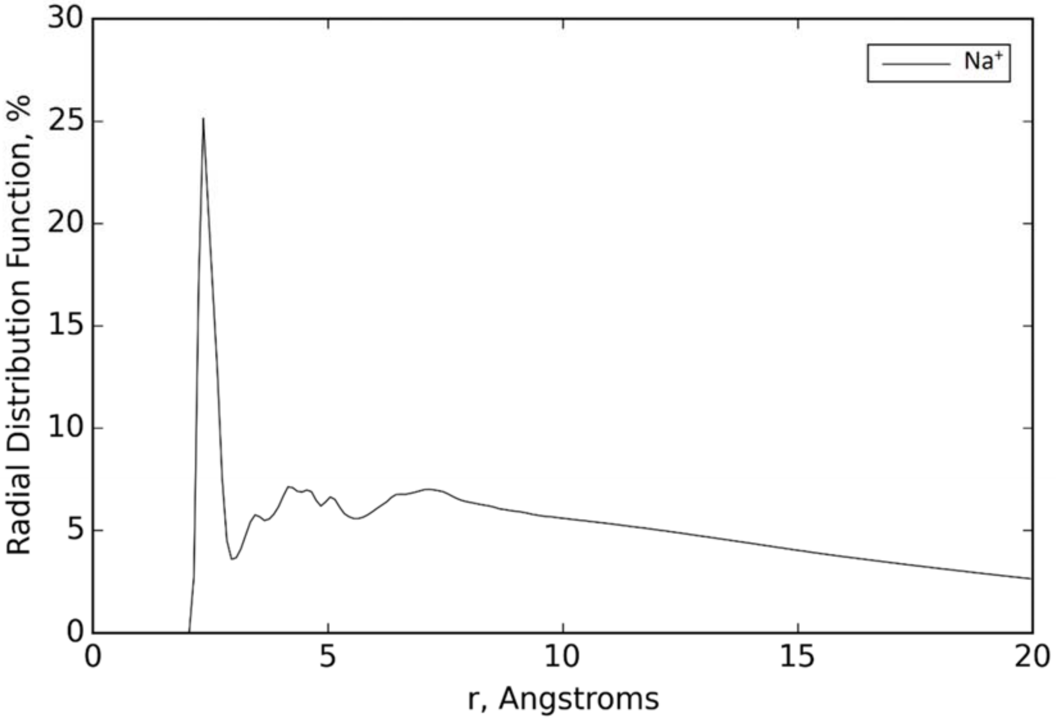

Supplement: Supplemental Materials [file NIHMS883851-supplement-Supplemental_Materials.pdf]
